# The Pan African Medical Journal 2015 - A year in review

**DOI:** 10.11604/pamj.2016.23.47.8934

**Published:** 2016-02-19

**Authors:** Raoul Kamadjeu

**Affiliations:** 1 Managing Editor, the Pan African Medical Journal, Nairobi, Kenya

**Keywords:** Publishing, open access, Africa, medical journal

## Brief

Time is accelerating; 2015 came and went by. It doesn't seem so long ago when we were celebrating the first article published in the Pan African Medical Journal (PAMJ) in mid-2008. It is a new year, an opportunity to reflect on our hits, misses and future plans. This report looks into just that. Academic publishing continues its rapid transformation with the rapid development of the open access publishing model. In 2009, it was estimated that 17% of the world’s articles were published in fully open-access journal [1]; a percentage surely higher in 2015. Open access publishers and journals are mushrooming at unprecedented speed, fueled by various motives, legit and predatory; unfortunately the status of biomedical publishing in Africa remains grim; open access or not. A study published in 2009 by Dirk Schoobaert in the Journal of Medical Librarian Association showed that only 38 of the 5000 journals indexed in Medline were from Africa [2]. A quick analysis of the Directory of Open Access Journal (DOAJ) show that, in January 2016, 771 (7%) of their 11129 journals are from Africa out of which 577 journals were from Egypt alone [3]. Although the absolute number of journals from African countries has increased in DOAJ over time, the contribution of Africa to the open access movement remains very small. It is in this fast changing and highly competitiveenvironment that we sailed through 2015.

### PAMJ in numbers

**Figure 1:**
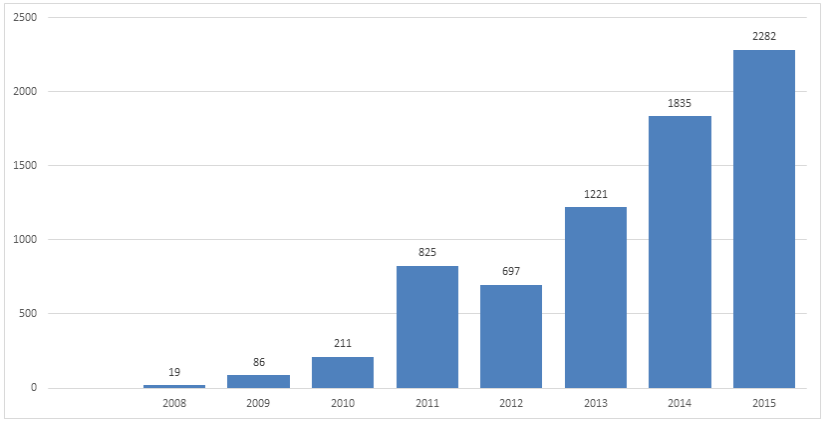
Trend in manuscript submission, Pan African Medical Journal, 2008-2015

**Figure 2:**
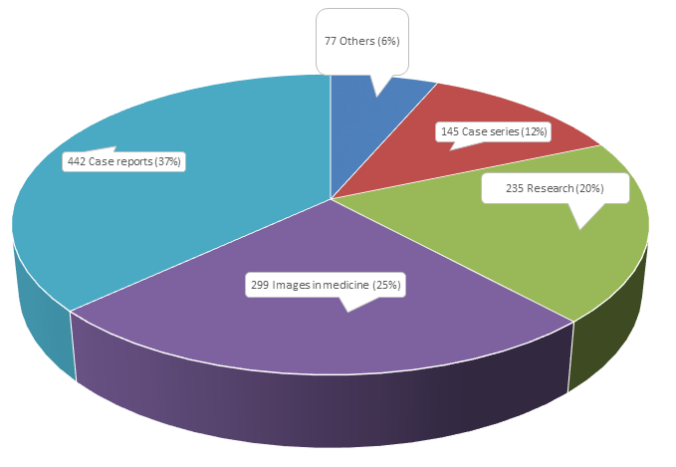
Manuscripts published by categories, Pan African Medical Journal, 2015

Since the publication of its first article in July 2008, PAMJ has experienced a rapid increase in the number of manuscripts received and published. 7922 articles have been submitted for consideration since Jul 2008 (Figure 1). In 2015, 2282articles (982 (43%) in English and 1297 (57%) in French), from 61 countries,were submitted for consideration; 1198 articles were published the same year (39% and 61% in English and French respectively). The dominant manuscripts categories in 2015 were Case Reports, Images in Medicine, Research and Case Series (Figure 2). Images in Clinical Medicine are becoming the fastest growing manuscript category submitted to PAMJ; with an average increase of more than 400% every year since 2012; this increase has to do with the widespread availability of camera on mobile phones and other mobile devices, giving clinicians the opportunity to share their bizarre, unique but educational encounter from the consultation room with their colleagues around the world. In the long term we hope to establish a valuable, practitioner-generated library of medical iconographies useful for clinical education. Close to 2/3 of manuscripts submitted in the categories Image in Clinical Medicine and Case Report originated from only two countries; morocco and Tunisia highlighting the quality of the technical facilities in the countries and the eagerness of their young practitioners to share their experience. These two countries were virtually absent from published research in 2015, which was dominated mainly by Nigeria, Cameroon, The Democratic Republic of Congo and Kenya (Table 1). 2015 was also the year we scaled up our human resources in order to cope with the increasing popularity of the journal. The PAMJ Cameroon Office which was officially opened in 16 December 2013 in Yaoundé with three full staff now hosts 7 associate editors. With the West (Cameroon) and East (Uganda) Africa offices operational, PAMJ is moving towards its goal of building editorial capacities and transferring modern editorial technology to the continent.

**Table 1:** Top 10 countries of origins of manuscript submitted and published in 2015

Submitted in the category Image in Clinical medicine and Case report		Published in the category Research	
Country	N (% of total submissions)	Country	N (% of total publications)
Morocco	432 (61%)	Nigeria	39 (17%)
Tunisia	106 (15%)	Cameroon	25 (11%)
Turkey	27 (4%)	Congo DR	21 (9%)
Senegal	27 (4%)	Kenya	17 (7%)
France	18 (3%)	Morocco	17 (7%)
Congo DR	13 (2%)	Ethiopia	16 (7%)
Burkina Faso	11 (2%)	Tanzania	10 (4%)
Nigeria	9 (1%)	Burkina Faso	9 (4%)
India	9 (1%)	Uganda	9 (4%)

### What is in store for 2016?

Improving the overall quality of the journal and authors experience remain our highest priority. This will be achieved through systematic adherence to high editorial standards. From January 2016, manuscripts in French will now include an English translation of the title, keywords and abstract provided by authors prior to publication; this is an effort to expand the reach of French manuscript. It is also our hope that 2016 will also see several of our projects come to fruition including the launch of our: a) Clinical Quiz a continuous medical education quiz derived from case reports and images published in PAMJ, which will allow clinicians to challenge their knowledge in clinical medicine; b) the PAMJ Conference Management System; an advanced online module to support the organization and management of scientific conferences in Africa. Training remains at the core of PAMJ strategy to help build a generation of African researchers fluent in reporting science. So far, an estimated 120 young researchers in Uganda, Kenya and Ethiopia attended workshops on some aspects of scientific writing organized by PAMJ and its partner AFENET; 2016 will see the launch of PAMJ University; a mechanismto help build the capacities of an increasingly large cohort of young African researchers in the fundamentals of reporting science.
